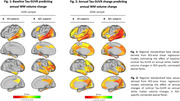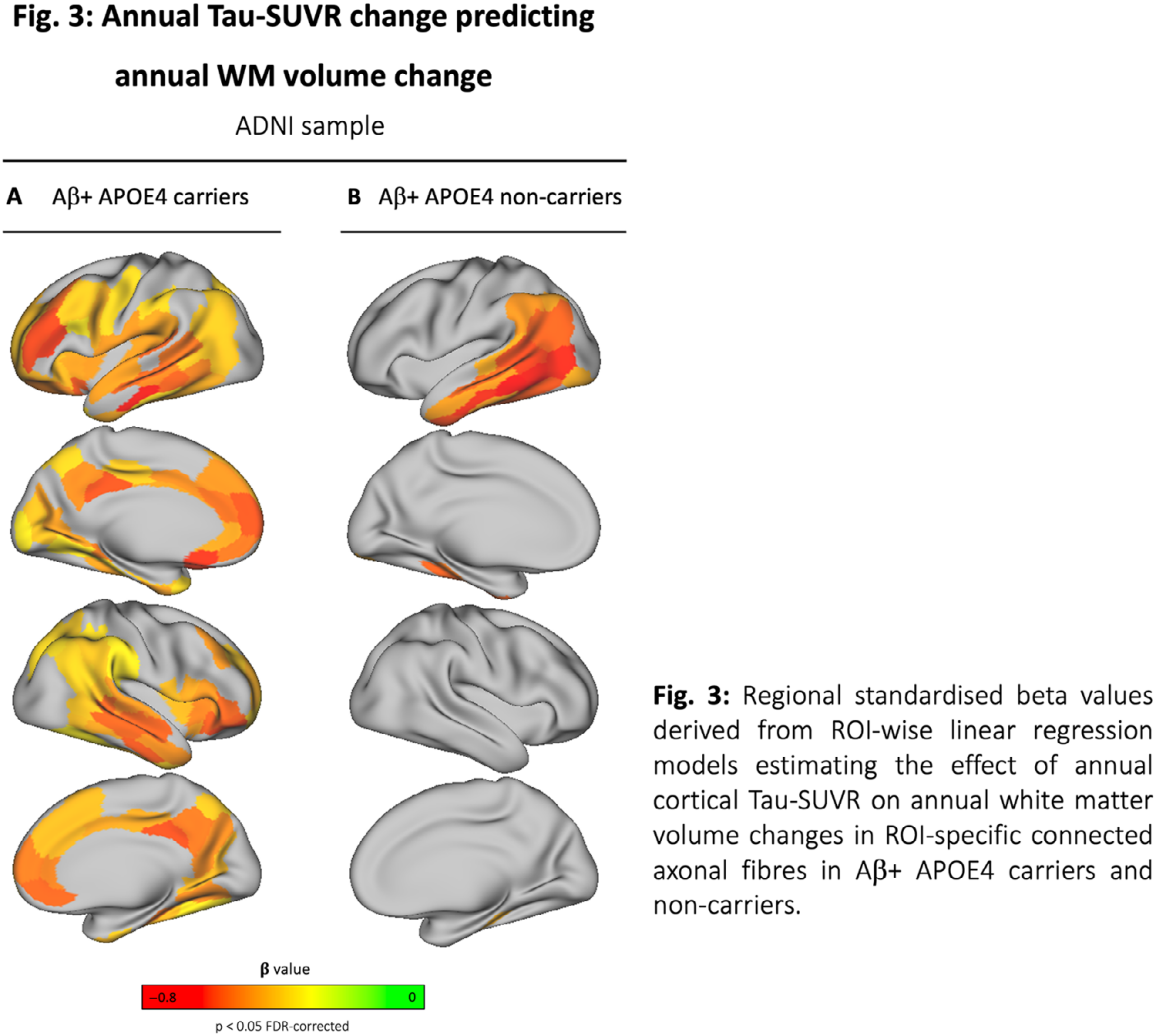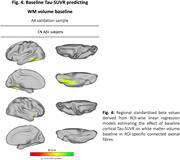# Cortical tau aggregation drives axonal degeneration in white matter fiber tracts in Alzheimer’s disease

**DOI:** 10.1002/alz.092399

**Published:** 2025-01-09

**Authors:** Julia Pescoller, Anna Dewenter, Anna Steward, Amir Dehsarvi, Fabian Wagner, Mattes Gross, Zeyu Zhu, Michael Ewers, Sebastian Niclas Roemer, Matthias Brendel, Nicolai Franzmeier

**Affiliations:** ^1^ Institute for Stroke and Dementia Research (ISD), LMU University Hospital, Munich, Munich (Bavaria) Germany; ^2^ Institute for Stroke and Dementia Research (ISD), University Hospital, LMU, Munich, Bavaria Germany; ^3^ Institute for Stroke and Dementia Research, LMU, Munich, Munich Germany; ^4^ German Center for Neurodegenerative Diseases (DZNE), Munich Germany; ^5^ Institute for Stroke and Dementia Research (ISD), University Hospital, LMU, Munich Germany; ^6^ Department of Neurology, University Hospital, LMU, Munich, Bavaria Germany; ^7^ Department of Nuclear Medicine, University Hospital, LMU Klinikum, Munich, Bavaria Germany; ^8^ Munich Cluster for Systems Neurology (SyNergy), Munich, Bavaria Germany; ^9^ University of Gothenburg, The Sahlgrenska Academy, Institute of Neuroscience and Physiology, Psychiatry and Neurochemistry, Gothenburg Sweden; ^10^ Institute for Stroke and Dementia Research (ISD), University Hospital, LMU, Munich, Bayern Germany

## Abstract

**Background:**

In Alzheimer’s disease (AD), cortical tau aggregation is a strong predictor of cortical brain atrophy as shown by MRI and PET studies, particularly driving the degeneration of neuronal somata in the grey matter. However, tau’s physiological role is to stabilize microtubules within axons in the brain’s white matter (WM) pathways. Therefore, tau’s white‐to‐grey‐matter translocation and aggregation in neurofibrillary tangles close to neuronal somata may induce WM degeneration through destabilization of axonal microtubule integrity. To address this, we determined whether cortical tau predicted faster atrophy of connected WM tracts in AD.

**Method:**

We included from ADNI cohort 37 amyloid‐PET negative (Aβ‐) cognitively normal (CN) participants and 88 amyloid‐PET positive (Aβ+) participants across the AD‐spectrum (i.e. CN/MCI/Dementia = 50/28/10), with baseline amyloid‐PET, longitudinal tau‐PET and longitudinal structural MRI data. For replication, we included baseline amyloid‐PET, tau‐PET and MRI data of 321 CN‐Aβ+ subjects from the A4 cohort. T1‐weighed MRIs were segmented into grey and white matter and non‐linearly normalized to MNI space using CAT12. The cortical Brainnetome Atlas and a diffusion imaging‐based tractography atlas were applied tau‐PET and MRI data to i) determine cortical tau‐PET accumulation rates within Brainnetome ROIs, and ii) assess WM volume changes within fiber tracts connected to each cortical ROI. Statistical regression‐models were adjusted for age, sex, WM hyperintensity volume, global amyloid, intracranial volume, and APOE4‐status.

**Result:**

In ADNI, higher baseline temporo‐parietal tau‐PET predicted faster volume reductions in connected WM tracts (Figure 1A), especially pronounced in Aβ+ subjects (Figure 1B). Similarly, faster tau accumulation was strongly linked to widespread WM volume reductions in connected fiber tracts (Figure 2A; Figure 2B), particularly exacerbated in Aβ+ APOE4 carriers (Figure 3A) compared to non‐carriers (Figure 3B). These results suggest that tau accumulation and WM degeneration are parallel processes in AD, modulated by APOE4. In the A4 validation sample of preclinical AD patients, we detected congruent associations between inferio‐temporal tau‐PET increase and reduced WM volume in connected fiber tracts (Figure 4).

**Conclusion:**

Cortical tau aggregation is associated with progressive WM atrophy in connected fiber tracts throughout the brain, suggesting that tau accumulation triggers axonal degeneration, which may induce neuronal disconnection and dysfunction and thereby contributing to AD progression.